# Supplementary material for: Intravenous antibiotics in preterm infants have a negative effect upon microbiome development throughout preterm life
Source: Gut Pathog. 2023 Apr 21;15:18. doi: 10.1186/s13099-023-00544-1 (PMC10120188; doi:10.1186/s13099-023-00544-1)
Supplement: Supplementary file 1 — Additional file 1. Methodological protocols. [file 13099_2023_544_MOESM1_ESM.docx]

# Methodological Protocols

## Sample collections

### Materials for stool sample collection and storage

1. 30ml universal container with spoon; polystyrene blue screw cap; printed label; sterile (Elkay Laboratory Products Ltd, UK)
2. 7ml polystyrene bijou container; with label; sterile (Starlab, UK)
3. Refrigerator at 4°C
4. Ultra-low temperature laboratory freezer at -80°C

### Method for stool collection and storage

Stool samples were collected from birth up to the cessation of the subjects’ participation in the study. One sample was requested per day (when available), and was obtained by the nurses caring for the baby, following the spontaneous passage of stool into the nappy, or, if indicated by the clinical team, assisted by the administration of a Glycerin suppository. As part of routine clinical care, preterm infants’ nappies are checked a minimum of four hourly, ensuring all samples were obtained within four hours of passage. Samples were removed from the nappy and placed into a sterile container using a sterile spoon, labelled with subject identity and date of sampling, and immediately placed into a designated 4°C refrigerator on the neonatal unit. These samples were subsequently processed by members of the research team, by transferral to a sterile bijou bottle, and relabelled with the subjects’ pseudo-anonymised subject identification code, the date of sampling, and sample identification code. The samples were subsequently transferred to an ultra-low temperature freezer within Homerton University Hospital for medium-term storage at -80°C, generally within 24 hours. There is inconsistency across the literature regarding the effect of storage temperature upon the accuracy of downstream microbiome data, but there is evidence to suggest that this duration of storage at 4°C is unlikely to contribute significantly to any variation in microbiome profile, and indeed the consistency of this methodology throughout the study contributed to its validity, and is compatible with the pragmatic approach required in an active clinical area.

### DNA Extraction

#### Materials for DNA extraction

1. Ice
2. 5μL inoculation loops
3. Tris-EDTA buffer
4. Eppendorf tubes
5. 20mM propidium monoazide
6. Laboratory vortex
7. 500W light
8. Small polystyrene tray
9. Pasteur pipettes
10. Microcentrifuge (Heraeus Biofuge Pico, Germany)
11. Vortex Adaptor tube holder (Qiagen, Netherlands)
12. Refrigerator at 4°C
13. Adjustable volume pipettes and tips
14. DNeasy® PowerSoil® Kit

- Powerbead tubes
- Solution C1 (detergent and disruption agents)
- Solution C2 (Inhibitor Removal Technology®)
- Solution C3 (Inhibitor Removal Technology®)
- Solution C4 (high concentration salt solution)
- Solution C5 (ethanol-based wash)
- Solution C6 (10mM Tris)
- Spin column

#### Methods for DNA extraction

##### Sample preparation

1. Samples were processed in batches (n=24). Samples were randomly allocated between batches (and subsequently, PCR plates and sequencing runs).
2. The batch to be processed was removed from the ultra-low temperature freezer, and defrosted on ice.
3. Each sample was homogenised using a 5μL inoculation loop, to maximise a uniform distribution of organisms, and to reduce ‘clumping’, which may prevent downstream permeation of the samples with propidium monoazide dye and/or Powerbeads.

- where the sample was too solid for homogenisation, 50μL of tris-EDTA buffer was added to the sample to facilitate this through emulsification.

1. 20mg of homogenised stool was added to an Eppendorf tube, and suspended in 500μL of Tris-EDTA buffer.

##### Use of propidium monoazide

In view of the findings of a pilot study assessment into the effectiveness of PMA dye, the following were carried out as per the manufacturer’s protocol:-

1. 1.25 μL of 20mM PMA (Biotium, USA) was added to each Eppendorf tube of stool suspension and vortexed for five seconds.
2. This mixture was incubated in the dark for five minutes, to allow the PMA to permeate through the compromised membranes of non-viable cells.
3. Thereafter, the PMA/stool mixture was exposed to a 500W light at a distance of 20cm for ten minutes, to activate the PMA dye. The samples were gently mixed and turned after 5 minutes, to maximise exposure (and thus activation) of the PMA; and treated on ice, to prevent overheating from the powerful overhead light.
4. Following this treatment, the Eppendorf tubes were centrifuged at 16060×*g* for two minutes, and the supernatant extracted and discarded (removing excess unbound PMA, prior to the cell lysis steps). The residual pellet was retained for subsequent DNA extraction.

##### Extraction protocol

The DNA was extracted using a modified protocol for the DNeasy® PowerSoil® Kit (Qiagen, Netherlands). In order to prevent differential rates of contamination from DNA/organisms within the extraction kit (*i.e*. “the kit-ome”), all extraction kits were ordered in advance and originated from the same batch.

1. The retained pellet was added to the Powerbead tube, and briefly vortexed, to homogenise the stool suspension and Powerbead/buffer mixture.

- a negative extraction control was also prepared for each sequencing run (four in total), where no sample pellet was added, and the protocol was followed as standard from there on (essentially containing extraction reagents only).

1. 60μL of Solution C1 was added to the Powerbead tube, and briefly vortexed, to further mix the suspension.
2. The 24 Powerbead tubes in the batch were affixed to the Vortex Adaptor, and agitated at maximum speed for 15 minutes.

- the combination of the physical agitation alongside ceramic Powerbeads, and mixing with detergents and disruption agents, accomplishes the dual effect of mechanical and chemical disruption of bacterial cells, to liberate the bacterial DNA therein for further downstream analysis.

1. The Powerbead tubes were centrifuged at 10000×*g* for 30 seconds, and the supernatant (containing dissolved bacterial DNA) extracted and transferred to a clean Eppendorf tube.
2. 250μL of Solution C2 was added to the Eppendorf tube from step 4, briefly vortexed, and incubated at 4°C for five minutes.

- solution C2 will precipitate residual non-DNA material which may inhibit downstream processing.

1. The Eppendorf tube from step 5 was centrifuged at 10000×*g*, and up to 600μL of the supernatant (with the pellet containing precipitated inhibitors) transferred to a clean Eppendorf tube.
2. 200μL of Solution C3 was added to the Eppendorf tube from step 6, briefly vortex, and incubated at 4°C for five minutes.

- solution C3 will further precipitate any residual inhibitory material not removed during step 5.

1. The Eppendorf tube from step 7 was centrifuged at 10000×*g,* and up to 750μL of supernatant transferred to a clean Eppendorf tube.
2. 1.2ml of Solution C4 was added to the Eppendorf tube from step 8, and briefly vortexed, to mix
3. 675μL of the mixed solution from step 9 was loaded onto a spin column, centrifuged at 10000×*g* for 1 min, and the filtrate discarded.

- the concentrated salt solution (C4) facilitates binding of the extracted DNA to the silica filter within the spin column, allowing the bacterial DNA to be selectively retained on the silica membrane, and remaining contaminants and reagents to be filtered through.

1. Step 10 was repeated two further times (retaining the spin column between filtrations), until all the mixed solution from step 9 has been filtered (and all the DNA bound to the silica membrane).
2. 500μL of solution C5 was added to the spin column, centrifuged at 10000×*g* for 30 seconds, the filtrate discarded, the spin column centrifuged again at 10000×*g* for 30 seconds.

- the alcohol wash solution (C5) selectively removes any remaining residual material upon the silica membrane, whilst leaving the DNA bound.

1. The silica membrane (containing the bound DNA) was removed from the spin column and placed in a clean Eppendorf tube.
2. 100μL of solution C6 was placed onto the spin filter, centrifuged at 10000×*g* for 30 seconds, and the filtrate (purified DNA solution) retained for subsequent processing.

- the addition of the elution buffer (C6) lowers the salt concentration at the silica membrane – DNA interface, permitting release of the DNA from binding, so that it may be filtered through the membrane to be retained in purified form in the filtrate.

### PCR Amplification of extracted bacterial DNA

#### Materials for PCR amplification

1. UV steriliser
2. Fusion primers (Eurofins, Germany)

- v4.SA501-508, v4.SB501-508

- v4.SA701-712, v4.SB701-712

1. Standard laboratory freezer at -20°C
2. Microcentrifuge (Heraeus Biofuge Pico, Germany)
3. Eppendorf tubes
4. Tris-EDTA buffer
5. Adjustable volume pipettes and tips
6. Laboratory vortex
7. Tris-HCl buffer
8. PCR plates
9. PCR water
10. Thermo Scientific Phusion Green Hot Start II High-Fidelity PCR Master Mix (Thermofisher, USA)
11. PCR gradient thermal cycler (Techne, UK)
12. Positive control (*Porphyromonas* *gingivalis)*
13. Microbial Community DNA Standard (ZymoBIOMICS, USA)

##### Materials preparation

PCR amplification of the 16S V4 hypervariable region was undertaken by means of a dual-indexing strategy, whereby the use of combinations of barcode-labelled forward and reverse primers allowed subsequent identification of the 16S amplicons from up to 384 (16×24) samples, from only 40 (16+24) fusion primers.

The fusion primers consisted of five parts:

- A MiSeq adaptor sequence (forward: i5; reverse: i7) to facilitate hybridisation of the amplicon to the sequencing flow cell oligonucleotides.
- A barcode (unique nucleotide sequence) to allow linkage between the amplicons and the originating sample throughout the sequencing process.
- A primer pad, to equalise the annealing temperatures of the forward and reverse primers
- A linker sequence, an anti-complementary segment to prevent interaction between the preceding (non-complementary) adaptor, barcode and primer pad segments with the target DNA.
- The primer, to permit subsequent clonal amplification of the 16S V4 hypervariable region.
  1. Under strict sterility and non-contamination precautions (UV sterilisation of all materials), the fusion primers (Eurofins, Germany) were prepared according to the manufacturer’s protocol.

- 40 Eppendorf tubes containing 150μL solutions of the 40 fusion primers (10μM) in Tris-EDTA/Tris-HCl were produced (sufficient for 4× PCR plates).

- 1. Using similar precautions as in step 1, the Thermo Scientific Phusion Green Hot Start II High-Fidelity PCR Master Mix (Thermofisher, USA) was prepared according to the manufacturer’s protocol, with 1.25ml of Master Mix dissolved in 0.95ml PCR water.

- the Master Mix contains the DNA polymerase; Affibody™ protein (a reversibly-bound inhibitor of DNA polymerase activity at ambient temperature); nucleotides; reaction buffer; and a density reagent and tracking dye for subsequent gel electrophoresis.

##### PCR amplification

1. These components were subsequently prepared for processing, with the following combined in a fresh PCR plate, sealed and mixed:

- 22μL of the Master mix /PCR water solution (12.5μL of Master Mix; 9.5μL of PCR water (from step 2)

- 0.5μL of forward primer

- 0.5μL of reverse primer

- 2μL of sample DNA /positive control (*P. gingivalis*)/negative extraction control/microbial community DNA standard – “mock community” (ZymoBIOMICS, USA)/negative PCR control (PCR grade-water)

This gives a total volume of 25μL per PCR plate well.

1. The PCR plate was run under the following thermo-cycling parameters:

- 2 minutes at 98°Cs (enzyme activation)

- 25 cycles of 98°C for 10 seconds (denaturation), 55°C for 30 seconds (annealing), and 72°C for 30 seconds (extension)

- a final extension step of 72°C for five minutes

1. The generated PCR products were stored at -20°C

### Gel electrophoresis of amplified 16S V4 bacterial DNA

#### Materials for gel electrophoresis of amplified 16S V4 bacterial DNA

1. Agarose
2. 0.5X TBE buffer
3. Duran flask
4. Laboratory microwave
5. Adjustable volume pipettes and tips
6. GelRed dye
7. Electrophoresis tray and combs
8. 100bp DNA electrophoresis ladder (New England Biolabs, USA)
9. UV camera

#### Methods for gel electrophoresis of amplified 16S V4 bacterial DNA

##### Preparation of 1% agarose gel

1. 0.5g of agarose was dissolved in 250ml of 0.5X Tris-borate-EDTA (TBE) buffer, and microwaved for one minute to dissolve.
2. Once cooled, 0.5μL of GelRed was added and gently mixed.

- GelRed is a nucleic acid stain, and when bound to DNA and exposed to UV light, will fluoresce, allowing visualisation of the electrophoretic bands.

1. The gel was poured into an electrophoresis tray, combs inserted and left to set.

##### Performing the gel electrophoresis

1. 2μL of the amplicons were inserted into each well, alongside a PCR water negative control and a 100bp DNA ladder.
2. The electrophoresis gel was set to run at 100V for 15 minutes, and subsequently read on a UV camera and GeneSnap software (Syngene, India).

- Where samples did not demonstrate the presence of a clear band at ~250bp, they were excluded from further processing and analyses.

### Normalisation of DNA abundance in samples

#### Materials for normalisation

1. SequalPrep™ (96 well) Normalization Plate (Invitrogen, USA)
2. SequalPrep™ Normalization Binding Buffer (Invitrogen, USA)
3. Adjustable volume pipettes and tips
4. SequalPrep™ Normalization Wash Buffer (Invitrogen, USA)
5. SequalPrep™ Normalization Elution Buffer (Invitrogen, USA)
6. Eppendorf tubes

#### Methods for normalisation

The normalisation step was carried out following the protocol for the SequalPrep™ Normalization Plate (96) Kit (Invitrogen, USA).

1. 20μL of PCR amplicons from each sample was placed in each well of the normalisation plate (in batches of 96).
2. 20μL of binding buffer was added to each well, and the solutions gently mixed.
3. The plate was left for one hour, to allow binding of the target amount of DNA (~25ng/well) to the plate surface.
4. After this period, the excess liquid was aspirated from each well, and discarded

- this contains a mixture any excess sample DNA and binding buffer

1. 50μL of wash buffer was added to each well, mixed, aspirated and discarded

- this removes any residual material which is not bound to the well

1. Removal of wash buffer was further maximised by inverting the normalisation plate, and tapping it onto paper towels, to physically shake out any residual wash buffer.
2. 20μL of elution buffer was added to each well, gently mixed, and allowed to incubate for five minutes.

- this allows the bound DNA to unbind and come into solution for further use.

1. 5μL from each eluted sample was aspirated, and pooled in a single Eppendorf tube

- this resulted in 16 Eppendorf tubes of pooled samples (16 separate PCR plates, to be processed through 4 sequencing runs)

### Quantitation of DNA in pooled samples

#### Materials for DNA quantitation

1. TE Buffer (10mM Tris-HCl, 1mM EDTA)
2. Autoclaved, distilled water
3. Quant-iT™ PicoGreen® dsDNA Reagent (Invitrogen, USA)
4. PCR plate
5. Lambda standard DNA (Invitrogen, USA)
6. Adjustable volume pipettes and tips
7. ClarioStar microplate reader (BMG Labtech, Germany
8. Tris-HCl buffer

#### Methods for DNA quantitation

The quantitation step was carried out following the protocol for Quant-iT™ PicoGreen® dsDNA Reagent (Invitrogen, USA).

##### Materials preparation

1. The TE buffer was diluted in autoclaved, distilled water, as per the manufacturer’s protocol, to generate 20ml of a 1X TE buffer solution.
2. The Quant-iT™ PicoGreen® dsDNA Reagent was diluted 200-fold in 1X TE buffer (see step 1), as per the manufacturer’s protocol, to generate 8ml of solution

- this step was performed with minimal light contamination, due to the reagent being photo-sensitive and susceptible to degradation; and with a plastic vessel, due to the possibility of reagent adsorption onto glass.

1. The Lambda DNA standard (100μg/ml) was diluted 1:10 in 1X TE buffer (see step 1), as per the manufacturer’s protocol, to generate a solution at concentration 10μg/mL.
2. Serial dilutions (from which to plot the standard curve) were generated by adding 2μL of the 10μg/mL Lambda standard DNA to 198μL of 1X TE buffer, followed by sequential 50% dilutions in 1X TE buffer (100μL of the respective DNA solutions transferred into 100μL of 1X TE buffer), to produce standard curve points of 10ng/well, 5ng/well, 2.5ng/well, 1.25ng/well, 0.625ng/well, 0.3.ng/well, 0.156ng/well and 0ng/well.

- these were produced in triplicate, and the average fluorescence taken to derive the standard curve

##### Assay processing

1. Pools were analysed in triplicate, with the intention to use the average DNA concentration for subsequent calculations.
2. 1μL of the pooled DNA from each of the 16 PCR plates was added to 99μL of 1X TE buffer

- in triplicate, this led to (3 × 16 pools) 48 wells being assayed

1. 100μL of the Quant-iT™ PicoGreen® dsDNA reagent was added to each of the pooled sample wells and the standard curve derivation wells, and gently mixed.
2. The reaction plate was covered to prevent photo-reactive degeneration of the Quant-iT™ PicoGreen® dsDNA reagent, and left to incubate for five minutes.
3. The fluorescence within each well was subsequently measured using the ClarioStar microplate reader (BMG Labtech, Germany) using standard fluorescein wavelengths (excitation ~480nm; emission ~520nm).
4. The DNA concentration per well can be inferred by plotting the fluorescence of pooled samples on the standard curve derived from the standard Lambda DNA concentrations.
5. All pooled samples were subsequently diluted with Tris-HCl to match the concentration of the most diluted pooled sample.
6. Finally, the 16 pooled samples were further pooled into 4 Eppendorf tubes (one for each sequencing run), with the volume of pooled sample added to the tube weighted for the number of individual samples within that pool.

## Sequencing Analysis

Next generation sequencing produces large volumes of raw data, which must be quality‑filtered and processed prior to downstream analyses. This is computationally demanding, and requires algorithmic analysis pipelines in order to be efficiently accomplished. We elected to use the DADA2 pipeline, which employs novel approaches to error identification (specific to the Illumina platform), and to identification of sample composition, which are theoretically superior to contemporary pipeline. DADA2 and all ensuing programs were run using R statistics software on a 2015 iMac and 2015 MacBook Pro.

All samples were processed through this common pipeline prior to partitioning for separate analyses. However, due to run-specific error profiles, they were initially analysed in batches based upon the originating sequencing run, to improve the accuracy of error identification.

### The divisive amplicon denoising algorithm

Of the millions of sequences identified by next generation sequencing, many will be identical, most likely representing an origin from the same taxa. The majority, however, will differ, stemming either from truly different initial taxa, or originating from the same taxa, but where errors in the sequencing process may have induced differences in their sequence reads.

The DADA2 algorithm uses a repeating iterative process to partition the reads into separate amplicon sequence variants (ASVs), based upon two main factors:

- The presumption that the most abundant sequences are likely to represent true sequences (as it is improbable that a large number of identical reads would have occurred through independent errors)
- The probability of the difference in base call being true, based upon the type of base transition (*e.g.* A→C, C→G), and the quality score at that particular base. These probabilities are calculated by a machine-learning algorithm, through iterative comparison of estimated error rates, and inference of sample composition within the dataset.

Using this methodology, sequences likely originating from unique taxa were identified and retained, and sequences likely emanating from sequencing errors discarded. In this dataset, standard DADA2 default parameters for the iterative algorithms were used.

### Merging paired reads

Up until this point, forward and reverse reads have been processed separately. DADA2 now allows the pairing of the denoised forward and reverse reads, by aligning overlapped forward and reverse sequences indexed to the same sample, creating contiguous sequences for further processing. This has the advantage of using the highest quality section of reads to inform the base call across the sequence, and double-checks the base-call assignation throughout the overlapped section. Any reads which did not align were discarded at this point. Following this merging step, the four sequencing run datasets (which had been processed separately) were merged into a common dataset.

Hereafter, the contiguous reads were reported as the forward sequence (read in a 5’ to 3’ direction), as is convention.

### Chimera removal

Chimeras are an artefactual product of PCR, where an inappropriately aborted extension product behaves as a mismatched primer in the next cycle, generating an amplicon composed of partial sequences from two different organisms. These are algorithmically removed by DADA2, by analysing the potential for sequences to be assembled from ‘left’ and ‘right’ segments of more abundant ‘parent’ sequences. Sequences clearly identified as chimeric were subsequently removed from the dataset.

### Assignation of taxonomy

By this point in the process, the unique ASVs within the dataset have been accurately identified, and their abundance within each individual sample determined. Whilst ASVs have particular value in their consistency and comparability between different studies, to translate findings to clinically utilisable information, the taxonomic label to which the ASV applies must be determined. DADA2 uses an exact matching methodology, which allows assignment of ASVs to taxonomic classifications down to species-level (where possible). This is accomplished by comparison of the ASV against a database of species-level reference sequences. Where there is a unique and unambiguous correspondence, the ASV is classified as originating from that species. Where an ASV matches to multiple reference sequences (as is not uncommon when assessing only a segment (V4) of the 16S gene), then that ASV is classified to the lowest taxonomic level which is common to all of the matched sequences (*i.e.* the 16S rRNA gene V4 region may be identical across multiple members of the same genus, with differentiating variability occurring elsewhere in the 16S gene). Finally, when a sequence does not unambiguously match to a reference sequence, DADA2 also allows classification to a taxonomic level using a naïve Bayesian classifier method. These methods were used to assign the ASVs derived from our dataset to taxonomic labels.

### Evaluation of accuracy

Within each sequencing run, a mock sample was included with known microbial composition, so that the accuracy of the sequencing, and subsequent processing and taxonomic assignment, could be evaluated. We used the ZymoBIOMICS Microbial Community DNA Standard (ZymoBIOMICS, USA) which contains DNA from eight species of bacteria (and two yeasts, which do not possess a 16S subunit gene, and would thus not be amplified). Following sequencing, the mock samples were identified, and their derived composition compared with the theoretical composition of the standard sample.

### Preparation for analyses

Following the quality control steps, the dataset was processed on the basis of sequence quality as far as the DADA2 protocol allows. At this point, the dataset was exported into the *phyloseq* package within R, to enable further processing and analyses.

### Removal of contaminant taxa

Despite the initial use of PMA to enable the removal of contaminant free DNA within samples at the start of extraction, the potential for the re-introduction of contaminant DNA persists throughout the remainder of the process, from extraction to sequencing. However, the use of negative control samples allows the identification of ASVs (and taxa) likely originating from contaminant organisms/DNA, rather than representing true constituents of the subjects’ samples.

The *decontam* package in R was utilised, which allowed the identification of taxa which were disproportionally found in negative control samples, so they may be selectively removed from the dataset. This package has the added functionality of allowing batched analyses, where differing sequencing runs are examined separately, as varying contamination profiles may be expected. The *decontam* package operates a null hypothesis that there is no difference in the prevalence of an ASV between samples and negative controls; and for the null hypothesis to be rejected, and an ASV classified as a true contaminant, it must be more prevalent in the negative control samples, with difference in prevalence having a *p*-value of <0.1 (default setting). However, there is the possibility of ASVs being mis-classified by this methodology, particularly where they have a genuine high abundance in just a small proportion of samples – consequently, manual assessment of potential contaminants was conducted prior to exclusion. After inspection of the ASVs classified as contaminants, it appeared that true ASVs were indeed being misclassified as contaminants, and consequently this threshold for significant difference in prevalence was lowered to *p*<0.05.

### Removal of samples

Before proceeding to analyses, samples where the read abundance was low were removed, as they may not have provided sufficient coverage to be representative of bacterial population from which the sample was obtained. Specifically, this may lead to underestimation of richness, and an inaccurate description of diversity. We chose a pragmatic cut-off of 5000 sequences/sample, and excluded those below this threshold from further analyses. This was in line with our group’s previous practice which has shown this has provided an adequate level of coverage, and in acknowledgement of the intended taxonomic analyses at family-level.

## Analyses

### Statistical Approaches

#### Biometrics

To quantitatively assess the progression of the microbiome, biometrics were employed which could numerically represent the wider microbiome, or components thereof.

The most frequently employed biometric within this work is alpha diversity, which was calculated, using the *vegan* package within R, as the inverse Simpson index (1/λ):-

$$1/ =1/\sum p_{i}^{2}$$

where *p_i_* is the relative abundance of an individual taxon *I* within the sample.

This particular biometric was chosen as it is influenced to a greater degree by the dominant components of a community (compared to the alternative commonly used biometric, the Shannon index); and it was the major constituents of the preterm gut microbiome which were the focus of this thesis. The *inverse* Simpson index (1/λ) was used instead of the standard Simpson index (λ) as it more intuitively increases as diversity increases (as opposed to the standard Simpson index).

In some analyses, the richness has been described, so that comparisons may be made with studies from the wider literature, where this metric is employed. However, in view of the limitations of this metric, this has not been used extensively, nor in comparative analyses.

To allow quantitative description of the constituent communities within the microbiome, the relative abundance of specific taxa is described, acknowledging the artefacts which may emerge with proportional measures such as this. These were calculated using the base statistical functions in R, alongside the *tidyr* and *dplyr* packages.

Changes in the composition of the microbiome during clinical interventions were also assessed with ordination techniques, using the *adonis* and *permutest* functions within the *vegan* package in R, to perform PERMANOVA and Permutation of Multivariate Homogeneity Of Groups Dispersions tests. Representative paired samples were identified from each subject spanning the start and end of a clinical intervention; an NMDS plot constructed, and the *DESeq2* R package used to identify taxa which differed significantly between the two states.

#### Distribution of data

It was noted in the preceding literature review that in describing the relative abundance of different taxa across a population, the underlying distribution of taxa within the population was rarely (if ever) accounted for. This is important, as the subsequent use of summary measures to describe the relative abundance of said taxa within a population may be misleading. Where the group distribution approximately follows a normal distribution across subjects, a summary statistic may be appropriate. However, when the underlying distribution is heavily skewed, or indeed bimodal, as has been frequently demonstrated for some taxa within the neonatal population, the use of summary measures will be misleading. Consequently, an assessment was made of the distribution of taxa across the population, before using summary measures to describe said population.

Choosing an appropriate representative measure of the relative abundance of taxa within a subject was complicated by the longitudinally evolving relative abundance of said taxa, with differing numbers of samples, assessed at varying time points. Reflecting the approach endorsed by Matthews *et al* in their paper **“**Analysis of serial measurements in medical research”**,** the area-under-the-curve (AUC) of relative abundance (calculated within the *DescTools* package, normalised for duration of study, was utilised – this is the recommended manner of summarising unevenly, irregularly sampled, peaked data (as generally seen in these subjects), and is a reasonable surrogate marker of central tendency of relative abundance.

Using the normalised AUC as a measure of relative abundance of taxa within a subject, the distribution of these values across the study population was assessed for congruence with a normal distribution, using the Shapiro-Wilk test. Where taxonomic abundances were seen to be reasonably normally distributed, the subsequent longitudinal progression of these taxa were described using summary statistics; where a normal distribution was not noted, summary longitudinal measures were not calculated, but the underlying pattern of distribution qualitatively described.

In the subsequent comparative analyses of longitudinal progression, the distribution of individual summary measures was assessed for correspondence with normality (again using the Shapiro-Wilk test); where the values were normally distributed, summary measures and parametric comparative analyses were employed; where the distribution of the underlying summary measures did not meet criteria for normality, appropriate descriptive statistics, and non-parametric statistical tests were employed.

#### Weighting

Statistical weighting was used in the derivation of summary values within this study – this is a technique which adjusts the contribution of data to the final summary value, relative to the underlying statistical strength of the raw data (*e.g.* a regression coefficient derived from 16 data points is more likely to lie closer to the true value, than a regression coefficient derived from only eight data points, thus is given greater weight in determining the group value), as outliers and experimental errors will have a proportionally reduced contribution to the final value. Weighting is used in three instances in this work:-

- In the derivation of individuals’ summary statistics, on the basis of number of sequences per individual sample (using the *deming* package).
- In the derivation of group summary statistics, on the basis of number of samples per individual (using the *survey* package).
- In accounting for twins/triplets included in the same analyses, on the basis of studies which have previously demonstrated some congruence between microbiome development in twin/triplet siblings. Where pairs of twins/triplets occurred in the same analyses, they were half-weighted to reflect their potential commonality.

#### Multiple comparisons

In view of another criticism raised within the literature review, regarding erroneous conclusions potentially being drawn after making inappropriate multiple comparisons analyses, care was taken to ensure this did not occur in this instance. This was of particular importance when assessing changes in constituent taxa, as the number of taxa identified in microbiome analyses (even at higher taxonomic levels) may be extensive.

The risk of multiple comparison errors was first mitigated by the pragmatic *post hoc* decision (although anticipated *a priori*) to describe relative abundance at a ‘family’ taxonomic level, as this represented the rank which allowed the highest degree of differentiation, whilst maximising the number of ASVs which could be successfully classified at that level (only 74.23% of ASVs were classifiable at genus-level, whereas 99.98% of ASVs were classifiable at family-level). Performing analyses at this higher taxonomic level ensured that fewer taxa would be analysed, thus reducing the risk of multiple comparison errors, although reducing the taxonomic specificity of the subsequent conclusions. The failure of more ASVs to unambiguously classify at genus-level is due to similarity within the 16S V4 region of differing species, which could not be differentiated below the family taxonomic level.

Thereafter, it was decided that only taxonomic families which had a median, normalised AUC (weighted by number of samples/individual) across the cohort of >1% would be considered for comparative analyses.

#### Longitudinal data

In view of the concerns that the methodological validity of some approaches in the wider literature may be hampered by multiple comparisons errors (when repeatedly sampling from subjects) – care was taken to maintain the legitimacy of the statistical approach to this longitudinal data. Further utilising the approaches espoused by Matthew *et al*, summary measures of individual longitudinal data were calculated and employed as raw data, to draw subsequent conclusions for groups. In the majority of the following analyses, in which apparent linear trends could be seen with time, this involved the derivation of a regression coefficient for the slope of an individual’s progression, and using summary measures of these individual slopes (*i.e.* median/mean coefficient of regression, depending on the distribution of the data) to produce a value which describes the group progression as a whole. Where appropriate, other summary measures (*i.e.* peak relative abundance, time to peak relative abundance) have also been described.

#### Regression analyses

After inspection of the pilot study data, and initial review of the main study data, it was noted that outliers from the general linear trend may adversely affect the accuracy of a simple linear regression, so the Theil-Sen estimator technique was used to derive a regression line through the data. This is a non-parametric approach which calculates the median slope connecting data points within a dataset, and is less sensitive to the effect of outliers. The data underlying the derivation of the Theil-Sen estimator was weighted by the number of sequences producing each individual sample’s diversity statistic, using the *deming* package.

#### Sample size

The use of quantitative longitudinal biometrics in microbiome studies of the preterm gut is rare: the study by Blakstad *et al* ( in their paper Enhanced nutrient supply and intestinal microbiota development in very low birth weight infants) used a repeated measures linear mixed model to derive longitudinal metrics similar to those employed in this work, and was the only such study identified in the systematic review taking such an approach. However, this study was only identified in the latter iterations of the literature review, having been published after recruitment had ceased and sequencing completed. Additionally, as with the majority of studies, the intermittent exposure to potential microbiome modulators had not been accounted for, meaning that the conclusions which could be drawn from these data were limited.

Analysis of the six stable subjects included within the pilot study allowed the estimation of the potential distribution of data within the wider study. Diversity was seen to progress at a mean rate of 0.37 units/week (weighted by both sequences/sample, and samples/subject). Notably, this was significantly elevated, compared to the value obtained by Blakstad *et al* (0.08 units/week), although the Blakstad cohort was not specifically selected for clinical stability. The weighted standard deviation within the pilot study of diversity progression within this small pilot study group was 0.27 units/week. It was difficult to ascertain the value of a clinically significant difference in means, following an intervention; Blakstad *et al* describe a greater than three-fold change in diversity progression following their intervention of enhanced nutrient provision. A pragmatic decision was made to ideally power the study to give an 80% power of detecting a 1SD difference in the rate of diversity progression between two groups (at a significant level of p<0.05), assuming a reasonably normal distribution of values: this would require 16 subjects in comparator groups. It is acknowledged that this modelling relies on data from a small pilot study group, assumptions regarding the distribution of data within the study cohort, and uncertainty regarding how large the difference in diversity progression need be to be clinically significant. As an absolute minimum, this study should provide these data for further studies. It should also be noted that in the absence of open-ended recruitment, and a lack of control regarding the allocation of participants to groups based upon clinical interventions, that these recruitment targets may not be met. However, these calculations do allow reflection on how ensuing results should be judged, dependent on the number of analysed subjects relative to these pre-specified statistical power requirements.
